# Scoliosis-Specific exercises can reduce the progression of severe curves in adult idiopathic scoliosis: a long-term cohort study

**DOI:** 10.1186/s13013-015-0044-9

**Published:** 2015-07-11

**Authors:** Alessandra Negrini, Maria Gabriella Negrini, Sabrina Donzelli, Michele Romano, Fabio Zaina, Stefano Negrini

**Affiliations:** ISICO (Italian Scientific Spine Institute), Via R. Bellarmino 13/1, Milan, 20141 Italy; Centro Fisioterapia Negrini, Vigevano, Italy; Department of Clinical and Experimental Sciences, University of Brescia, Brescia, Italy; IRCCS Fondazione Don Gnocchi, Milan, Italy

## Abstract

**Background:**

Scoliosis fusion surgery is generally considered the only means to stop the progression of adult idiopathic scoliosis (ADIS), but for patients refusing surgery there is lack of evidence in favour of conservative treatment. The aim of the present study was to verify the possible effectiveness of scoliosis-specific exercises when facing ADIS progression.

**Methods:**

We designed a retrospective cohort study.

We included 34 ADIS patients in treatment at our Institute (5 males and 29 females, mean age was 38.0 ± 11.0), exclusively treated with specific Scoliosis Specific SEAS exercises.

**Instrumentation:**

SEAS exercises are scoliosis-specific exercises. In adult patients they are aimed to recover postural collapse, postural control and vertebral stability through an active self-correction. Postural integration is a key element, including the neuromotor integration of correct postures and an ergonomic education program. Therapy includes at least two weekly exercise sessions each lasting 45 min.

**Outcome measures:**

Radiographic progression was the main outcome and it was analysed as a continuous variable.

**Statistics:**

One way ANOVA and paired *t*-test were applied for continuous data, while chi-square test was applied for categorical data. Alpha was set at 0.05.

**Results:**

The mean Cobb angle of the patients included into the present study, was 55.8 ± 13.2 °. Fifteen patients had previous x-rays testifying scoliosis progression: the average curve progression (worsening) was 9.8 ± 6.6 ° at a median of 25 (range 17–48) years. The remaining were characterized by more severe curves, exceeding 40 ° Cobb (mean curvature 50.9 ± 13.6) but it was not possible to prove that the curves had progressed in these cases. After an average period of 2 years of treatment (range 1-18y), 68 % of the patients experienced an improvement in their scoliosis. However in one patient (3 %) the scoliosis worsened by 5 ° in 18 years (progression rate reduced from 0.5 ° to 0.27 ° per year). Patients improved 4.6 ± 5.0 ° Cobb (P < 0.05), with no differences based on the localization of the curve, gender, age, length of treatment, Cobb degrees at the start of observation or treatment.

**Conclusions:**

Scoliosis Specific SEAS Exercises proved to be superior to natural history in ADIS, at least in individual cases and should be considered as a first line treatment especially in patients refusing scoliosis surgery.

## Background

It has long been known (since 1969) that idiopathic scoliosis can continue to progress during adulthood after skeletal maturity [1]. Its evolution is slow and insidious and involves both the anatomic and the functional aspect of the curve development or the worsening of painful spinal or radicular symptoms and/or decompensation [2]. Once asymmetric load or degeneration occurs, the pathomorphology and pathomechanism in adult idiopathic scoliosis (ADIS) is quite predictable. Asymmetric degeneration leads to increased asymmetric loading and therefore to a progression of the degeneration and deformity [3].

More than 60 % of cases of ADIS progress, particularly the curves exceeding 30 ° Cobb at skeletal maturity, regardless of the curve pattern [4, 5]. Marty-Poumarat [6] showed that the rate of progression in ADIS is linear, and it can be used to establish an individual prognosis. This rate of progression is deemed to be around 0.5-1 ° per year [3, 6]; together with the known radiographic measurement error, recognised in 5 ° [7, 8], this drives to the consequence that individual variations can rarely be ruled out before some years of observation.

Different types of treatment are available: scoliosis specific exercises, various type of spinal orthesis, soft or rigid and surgery. Surgery is generally considered to be the only intervention that can stop curve progression, while scoliosis specific exercises and orthesis are mainly considered for pain control [3, 4]. In the literature, the prevailing treatment is surgery [3]; but scoliosis specific exercises together with other type of physical treatment, such as manipulation program, has also been proposed to avoid or stop curve progression [11, 12]. In a short-term case series Weiss et al. showed 43.93 % of 107 patients improved 5 or more Cobb degrees immediately after 4–6 weeks of in-patient exercise program [9]. Morningstar et al. showed that 19 patients treated with spinal manipulation and various physiotherapic procedures reported immediately after the therapy an average correction of 17 ° Cobb [10]. In a case report, Negrini A et al. showed a 18.5 Cobb degrees reduction after one year of Scoliosis Specific SEAS exercises [13].

In Italy there is a long tradition of specific exercise prescription for idiopathic scoliosis, with recently proven results in adolescents [14–18]; when ADIS patients decide to try and avoid surgery, scoliosis-specific exercises have been proposed for many years at our centre. It is very important to investigate the evidence regarding different conservative alternatives within a reasonable timeframe. The aim of this study was to retrospectively review all the ADIS patients in treatment in our Institute, to ascertain the effectiveness of a scoliosis-specific exercise treatment that might limit the progression of severe curves in idiopathic adult scoliosis patients.

## Methods

### Study design

This was a retrospective cohort study, including all the ADIS patients in treatment in our Institute with Scoliosis Specific SEAS Exercises until October 2008.

### Participants

Inclusion criteria were: adults (18 years or more and Risser 5 stage achieved) with ADIS larger than 30° and documented curve progression during adulthood (at least 6 ° Cobb) or larger than 40 ° who had refused surgical treatment. Patients were prescribed Scoliosis Specific SEAS Exercises exclusively. Patients were assessed a minimum of 1 year after their first assessment. All patients had to practice their exercises regularly for at least ten months per year; X-rays after one year of therapy at least. De-novo scoliosis and fused patients were excluded [3].

We included two sub-groups of patients: one group included demonstrated progression (PP), the other group had high degree scoliosis but with no definitive proof of progression apart from the patients’ declaration (nPP). In both cases, patients adhered to treatment actively, declaring their willingness to try and avoid fusion.

### Outcome measures

To evaluate the effect of treatment, we considered the last available x-ray for each single patient. All x-rays were blindly measured twice by the same expert physician (SN) and the worst result was considered: in a previous study his repeatability error proved to be less than 3° Cobb [19].

Moreover, patients were categorized as unchanged, worsened, or improved according to both the intra-rater repeatability for Cobb angles measurements of the expert involved [20] and the classical 5 ° limits [8]. We considered results both in terms of the most important curve of each single patient as well as in terms of the mean of the curves included. We deemed patients changed if at least one of their curve’s progressed or improved. Statistical analysis: After applying the Shapiro-Wilks test to check for normality, one way ANOVA and paired *t*-test were applied for continuous data, while chi-square test was applied for categorical data. Alpha was set at 0.05.

## Results

Populations: 34 patients (29 females, 5 males; mean age 38.0 ± 11.0 years) out of 381 patients from our database fulfilled the inclusion criteria and were included in this study. Curves’ type rates are shown in Table 1.

**Table 1:** Curve’s type in the sample of subjects considered

Curves type		N	%
Single curve	TH	4	12 %
N = 14	TH-L	4	12 %
% = 41.2 %	LU	6	17 %
Double curve	TH + L	17	50
N = 20	TH + TH-L	1	3
% = 58.8 %	TH DOUBLE MAJOR	2	6

The worst curves of the 34 patients measured 55.8 ± 13.2 ° Cobb; results are shown in Table 2, upper part.

**Table 2:** Left part: Cobb degrees comparison in all participants and in the two subgroups considered. Right part: results according to curves’ type

		Patients						Curves									
		Age (yy.mm)		Worst		Average		Total		Proximal thoracic		thoracic		Thoracolumbar		Lumbar	
		Pre	Post	Pre	Post	Pre	Post	Pre	Post	Pre	Post	Pre	Post	Pre	Post	Pre	Post
Total	Average	38.02	41.03	55,8	51,1	51,6	47,5	50,7	46,7	42,5	42,0	52,9	48,0	49,0	46,0	49,4	45,8
	Standard Deviation	11.01	10.11	14,1	12,9	12,8	13,0	13,8	13,5	13,4	21,2	15,2	14,6	12,7	4,9	12,9	11,0
	*P*			<0.001		<0.001		<0.001		NS		<0.001		NS		<0.001	
PP	Average	40.04	44.02	55,1	50,6	52,5	48,2	50,4	46,2	42,5	42,0	54,2	49,6	61,5	57,5	44,8	40,3
	Standard Deviation	10.10	10.11	13,2	14,4	13,4	12,4	13,5	13,7	13,4	21,2	15,4	16,0	6,4	12,3	9,8	12,8
	*P*			<0.005		<0.005		<0.001		NS		<0.05		NS		<0.05	
nPP	Average	36.06	38.11	56,3	51,6	50,8	46,9	50,9	47,0	-	-	52,0	46,9	40,7	38,3	51,9	48,8
	Standard Deviation	12.04	12.03	14,1	14,0	10,7	10,4	13,6	13,5	-	-	16,9	15,8	7,8	7,8	10,5	11,0
	*P*			<0.001		<0.001		<0.001		-		<0.001		NS		<0.01	
P among sub-groups		NS	NS	NS	NS	NS	NS	NS	NS			NS	NS	<0.05	NS	NS	NS

A further analysis was performed to compare patients with demonstrated progression (PP group). Patients with severe curves, but without x-ray evidence of progression (nPP group): fifteen patients were prescribed exercise since they had a progression of 9.8 ± 6.6 ° Cobb during an average period of 25 years (range 17–48), but their curves were below 40 ° Cobb (PP group); while 19 patients with curves over 40 ° who decided to try to prevent progression with exercises belonged to then PP sub-group.

At the start of treatment, the average and worst curves did not show any overall difference among subgroups (Table 2); we found significant differences only for the Thoracolumbar curves magnitude in PP and nPP groups (61.5 ± 6.4 and 40.6 ± 7.8 respectively); regarding curves type in the PP subgroup there was a significantly higher rate of double curves and Lumbar curves (12 vs 5).

**Fig. 1:**
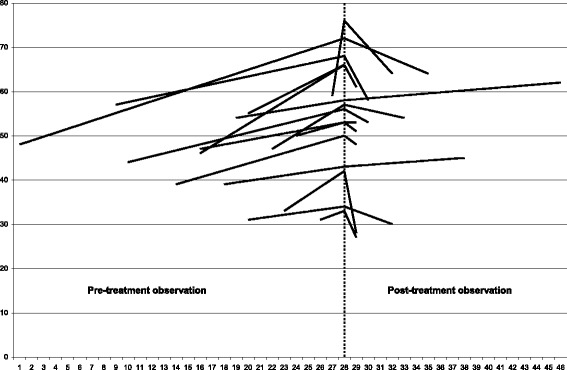
Results after about 2 years of therapy, according to the 3° Cobb degrees measurement error

After 2 years of therapy (range 1–18 years) 74 % of patients improved over the measurement error of 3 °, while 6 % (2 patients) progressed (Fig. 1); considering the “classical” threshold of 5 ° these percentages were 68 % and 3 % (1 patient) respectively (Table 3). In the nPP group there was a tendency toward better results (81 % improved and 5 % progressed vs 67 % and 7 % respectively, p = 0.07); we did not find any difference in terms of improvement/progression according to the localization of the curve, while there were differences among subgroups, mainly considering the 3 ° limits (Table 3).

**Table 3:** Results expressed in rate of improved, stabilized or progressed, based on the 5° Cobb threshold, promoted by the SRS

		Patients						Curves									
		*At least one curve*		*Worst*		*Average*		*Total*		*Proximal thoracic*		*Thoracic*		*Thoracolumbar*		*Lumbar*	
		3	5	3	5	3	5	3	5	3	5	3	5	3	5	3	5
Total	*Improved*	74 %	68 %	65 %	50 %	68 %	47 %	57 %	56 %	50 %	50 %	63 %	54 %	60 %	40 %	52 %	48 %
	*Stable*	19 %	29 %	32 %	48 %	29 %	53 %	37 %	44 %	0 %	0 %	33 %	46 %	40 %	60 %	43 %	52 %
	*Progressed*	6 %	3 %	3 %	2 %	3 %	0 %	6 %	0 %	50 %	50 %	4 %	0 %	0 %	0 %	4 %	0 %
PP	*Improved*	67 %	53 %	67 %	47 %	67 %	47 %	64 %	50 %	50 %	50 %	60 %	50 %	100 %	50 %	63 %	50 %
	*Stable*	27 %	40 %	27 %	53 %	27 %	53 %	27 %	45 %	0 %	0 %	30 %	50 %	0 %	50 %	38 %	50 %
	*Progressed*	7 %	7 %	7 %	0 %	7 %	0 %	9 %	5 %	50 %	50 %	10 %	0 %	0 %	0 %	0 %	0 %
nPP	*Improved*	81 %	81 %	63 %	63 %	68 %	47 %	53 %	50 %	-	-	64 %	57 %	33 %	33 %	47 %	47 %
	*Stable*	13 %	19 %	37 %	37 %	32 %	53 %	44 %	50 %	-	-	36 %	43 %	67 %	67 %	47 %	53 %
	*Progressed*	6 %	0 %	0 %	0 %	0 %	0 %	3 %	0 %	-	-	0 %	0 %	0 %	0 %	7 %	0 %
Chi square		<0.05	<0.001	<0.05	<0.05	<0.05	NS	<0.05	NS	NA	NA	<0.005	NS	NA	NA	<0.01	NS

The two patients who progressed (one per subgroup) had the following characteristics: in PP, one male with a Thoracic double major curve that started treatment at 28 years of age because of a previous progression in 8 years (from 52 °-54 ° to 52 °-58 °; rate of 0.5 ° per year): in the following 18 years of treatment he progressed to 57 °-62 ° (rate of 0.22-0.27 ° per year); in nPP, one female with a double thoracic and lumbar curve who started at 51 years and progressed in 5 years from 40 °-38 ° to 41 °-41 °.

All curves improved for all localizations, even if the less numerous groups (5 thoracolumbar curves, and 2 proximal thoracic) did not reach statistical significance; we did not find any statistical difference among groups for these results (Table 2).

We also did not find any statistically significant differences according to gender and age. Time is correlated with Cobb degree progression before the start of scoliosis specific exercises, while after the beginning of the treatment this correlation is lost.

We also included 5 patients who re-started treatment after an interruption of 6 years (range 3–10): in this period they had worsened from a minimum of 3 ° in 3 years to a maximum of 13 ° in 8 years. Two of them had a follow-up x-ray after the new period of Scoliosis Specific Exercises: one patient, previously progressed 8 ° in 10 years during the interruption, reduced the curvature of 8 ° in 9 years of exercises; the other, previously worsened 10 ° in 7 years, worsened 5 ° more in 21 years of treatment.

## Discussion

The present cohort study confirmed that when patients with adult scoliosis progress, Scoliosis Specific Exercises can be effective to obtain stability and in some cases to reduce the Cobb angles in degrees. In highly progressive curves, exercises appear to slow down the progression of the curvature (worsening). In addition, this study gives preliminary insight to the fact that continuity and consistency in Scoliosis Specific Exercises is mandatory, in order to not lose any improvements in results. For what concerns the analysis in the two subgroups PP, and nPP, the two groups were different in terms of curve morphology at start, and this could have affected the results. This may be one of the reasons for the tendency toward better results in the nPP sub-group. Nevertheless, the patients belonging to the nPP group, had more severe curves, usually considered good candidates to fusion independently from proven progression or not [3].

The interpretation of the results for the nPP group and the whole population could be considered as potentially biased, since these patients were stables when they entered the treatment. Moreover the pattern of curves was slightly different from the one described in long term follow up of scoliosis [21]. Nevertheless, since most of the patients improved, we considered this a valuable result.

**Fig. 2:**
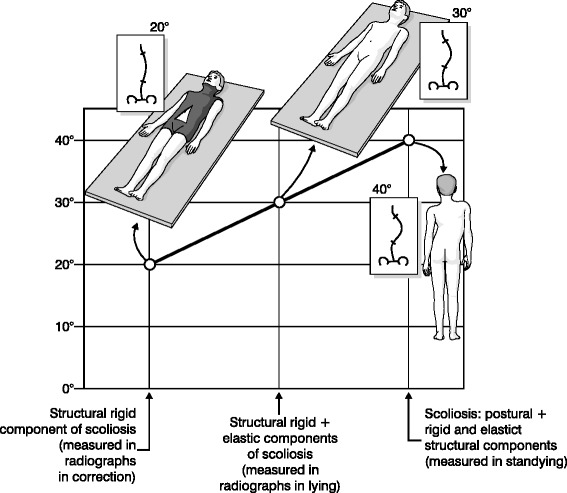
The postural component of scoliosis [19]. A scoliosis curve is made of many different components, including a postural one. Duval-Beaupére [19] described the case of three different radiographs: standing (SR), lying down (LR) and in correction e.g. using a cast (CR). The structural bony component can be measured with the CR; the structural ligamentous component comes from the difference between LR and CR; the postural component from the difference between SR and LR

There is an important question coming out from these results of improvement: how is it possible through “simple” scoliosis-specific exercises to obtain the reduction of scoliosis in ADIS? We do not have answers, but we do have a reasonable hypothesis. Duval-Beaupère [22] described the case of three different radiographs that, made at the same time in the same scoliosis patients, resulted in a curve magnitude that progressively decreased: standing (SR), lying down (LR) and in correction e.g. using a cast (CR) (Fig. 2). While only CR gives the fixed, not corrigible osteo-ligamentous deformity, the author describes the difference between SR and LR as a “postural collapse” that is the difference between the LR and CR values is seen as the “reducibility” due to concave ligament stretching. In other words, the classical SR gives the entire scoliosis, made up of some components including a postural one. This has also been quantified: in adolescents, regardless of curve magnitude. The mean difference between a standing radiograph and a supine one has been quantified as 9 ° Cobb [23], or around 20 % [24]. In ADIS it has been shown that in severe curves (mean Cobb angle: 60 degrees) performing an x-ray at different hours of the day [25] can give a measurement error due to the worsening of the curve as the days goes by ie from the morning to the evening: this can easily be attributed to the postural collapse of the spine. Accordingly, while the described reduction of scoliotic curve through SS exercises is certainly not due to a reduction of the bone deformity, it could easily be attributed or explained as the activity of the specific muscle acting either against gravity in standing or with gravity eliminated in lying down and a consequent recovery of the postural collapse, which is present in upright posture [26].

An anti-gravity correction can result in a long term achievement mainly through biomechanical means. While in adolescents a better growth of the vertebrae is allowed [27], in adults the process involved is bony progressive deformation due to degeneration. In ADIS postural collapse can have a role in enhancing progression, in the long term, due to the chronic asymmetric increase of compression. On the other hand, the reduction of the postural collapse (that is congruent with the reduction of the standing x-ray in the short term) can reduce asymmetric degeneration and consequently ADIS curve progression. Obviously, the increased quality of movement together with the biomechanical changes of the spinal soft tissues could play a role in decreasing the risk of progression [27, 28]. In conclusion when considering the importance of sagittal balance and its correlation with pain and disability [29–33] these results suggest that Scoliosis Specific Exercises can play a significant role in the management of patients with IS. Scoliosis Specific Exercises may enhance the possibility of compensation, thus preventing the loss of sagittal balance typical of the progression of scoliosis. |From this perspective, Scoliosis Specific Exercises could be effective in decreasing pain and disability resulting in an increased quality of life. It is hoped that future in depth studies will explore and analyse this important outcomes further. This cohort study opens up a new perspective to the approach to ADIS, particularly of patients that appear to progress. Instead of a fatalistic “wait and see” approach, an active one could be considered in the attempt to postpone, and eventually avoid surgery.

Even though this study presents a low level of evidence, current literature confirms the very short term effect of Scoliosis Intensive Rehabilitation (SIR) [9], SS exercises [13] and also manipulations [10, 11, 26]. In addition one case report showed good long term results with a multimodal treatment – exercises together with manipulative medicine [12]. Literature is however still lacking studies that compare different kind of Scoliosis Specific Exercises. Therefore in order to fill the gap in knowledge more studies are needed. Considering the very important challenges of conducting Scoliosis Specific Exercise RCT’s it is clear the importance of evidence of a lower level is needed to increase the knowledge base. From this point of view the main strength of the present study are the long term results, in a carefully selected population, that allows some inferences regarding Scoliosis Specific Exercise effectiveness to stop or reduce progression in adult scoliosis. The long period of follow up, limited the sample size; the convenience sampling is another limit that can affect results. In particular we can hypothesize that only patients refusing surgery and with an attitude towards the acceptance of actually doing the Scoliosis Specific exercises were included in this cohort which is a factor that can affect generalizability.

## Conclusion

Exercises proved to be superior to natural history in ADIS, and should be considered a possible tool for curve control. Future larger, long term, observational studies, should focus on defining the best Scoliosis Specific Exercises management approach and explore other very important issues associated with adult progressive spinal deformities, such as, sagittal global balance, back pain, disability and quality of life.
